# An Individual Differences Approach to Temporal Integration and Order Reversals in the Attentional Blink Task

**DOI:** 10.1371/journal.pone.0156538

**Published:** 2016-05-26

**Authors:** Charlotte Willems, Jefta D. Saija, Elkan G. Akyürek, Sander Martens

**Affiliations:** 1 Department of Neuroscience, University Medical Center Groningen, Groningen, the Netherlands; 2 Neuroimaging Center, University of Groningen, Groningen, the Netherlands; 3 Department of Psychology, Experimental Psychology, University of Groningen, Groningen, the Netherlands; University of Hyderabad, INDIA

## Abstract

**Background:**

The reduced ability to identify a second target when it is presented in close temporal succession of a first target is called the attentional blink (AB). Studies have shown large individual differences in AB task performance, where lower task performance has been associated with more reversed order reports of both targets if these were presented in direct succession. In order to study the suggestion that reversed order reports reflect loss of temporal information, in the current study, we investigated whether individuals with a larger AB have a higher tendency to temporally integrate both targets into one visual event by using an AB paradigm containing symbol target stimuli.

**Methodology/Principal Findings:**

Indeed, we found a positive relation between the tendency to temporally integrate information and individual AB magnitude. In contrast to earlier work, we found no relation between order reversals and individual AB magnitude. The occurrence of temporal integration was negatively related to the number of order reversals, indicating that individuals either integrated or separated and reversed information.

**Conclusion:**

We conclude that individuals with better AB task performance use a shorter time window to integrate information, and therefore have higher preservation of temporal information. Furthermore, order reversals observed in paradigms with alphanumeric targets indeed seem to at least partially reflect temporal integration of both targets. Given the negative relation between temporal integration and ‘true’ order reversals observed with the current symbolic target set, these two behavioral outcomes seem to be two sides of the same coin.

## Introduction

Only a tiny part of all available visual input can be perceived consciously. The process of selection happens through allocation of attention to relevant information that is present in our surroundings. Although this system of selective attention works relatively well in most situations, if two to-be-identified targets (T1 and T2) are presented in rapid temporal succession (200–500 ms), identification of T2 nevertheless frequently fails. This cognitive limitation is called the attentional blink (AB;[1]), a phenomenon that has allowed researchers to study the mechanism of temporal selective attention on the border of success and failure.

### Individual differences

Although the AB can be seen as fairly robust and universal [2], there are large individual differences in AB task performance with some individuals even showing no AB [3, 4]. Studying the occurrence and nature of these individual differences can be informative about the origin of the attentional strategy that is assumed to underlie the AB. That is, rather than a structural bottleneck, the AB has been suggested to be the result of applying an attentional strategy that is suboptimal for the AB task [5–8], where evidence points to a role of the distribution of attention deployed to the Rapid Serial Visual Presentation (RSVP) stream of stimuli, and the first target in particular [5, 8–14]. Various models have been proposed to address this attentional strategy assumed to underlie the AB. A common, implicit assumption is that our attentional system is optimally tuned for events at a more leisurely pace than that is required in AB tasks, in which it consequently falls short. For example, according to the threaded cognition model, the AB is due to overinvestment of attention to T1 processing, with T2 processing being postponed to protect the consolidation of T1 in working memory [8]. According to the competing boost and bounce model, attentional selection functions as a filter that causes irrelevant information to be suppressed, while relevant information is boosted [15]. Because these processes carry a slight time lag, in the AB task, T2 is accidentally suppressed, because the distractor following T1 was boosted. According to yet another current model of the AB, the episodic simultaneous type serial token (eSTST) model, the AB originates from a mechanism that provides episodic distinctiveness between WM representations [16]. Here, T2 is missed, because the items following T1 are suppressed to prevent interference between WM representations. While these and other models provide reasonable explanations in line with experimental findings, the exact nature of these seemingly adverse attentional strategies remain somewhat unclear. One way to further unravel the origin of the AB is by focusing on individual differences in AB task performance.

### Temporal integration

Earlier, investigating the temporal profile of individual differences in the AB, we have shown that individuals with a smaller AB magnitude show higher preservation of temporal order information, reflected in fewer reversed order reports of T1 and T2 at lag 1 [17]. That is, when T1 and T2 are presented in direct temporal succession, i.e., at lag 1, T2 accuracy is relatively high compared to other short lags. This is referred to as lag-1 sparing. However, at lag 1, temporal order information is more often lost, reflected in more reversed order reports of T1 and T2, than at later lags. Some researchers have subscribed these order reversals either to T2 being earlier consolidated than T1 [16] or to T2 entering the consolidation stage earlier than T1 [15], which will both result in T2 being reported before T1.

Another possibility, as suggested by Akyürek and colleagues [18], is that order reversals at lag 1 may at least partly be caused by a mechanism of temporal integration that merges separate visual events into one single representation. They demonstrated that in an RSVP where two targets could be integrated into one visual concept, i.e., where two symbol targets could be combined into another valid symbol target (e.g., “\” and “/” form “X”), temporal integration occurred regularly at lag 1. Furthermore, the number of order reversals decreased strongly compared to a paradigm where directly reporting integrated percepts was not possible, as is the case when using alphanumerical target stimuli. This link between order reversals and integrations confirmed the idea that the order reversals seen in classic AB tasks, in which targets can typically not be reported in a combined form, principally reflect a loss of temporal information that can be attributed to integration.

The temporal window in which these visual events are integrated was furthermore found to be adaptable [19, 20]. By varying stimulus presentation rate in a classic alphanumeric AB task, it was shown that the expectation of a slow presentation rate induced more temporal integration (measured indirectly with order reversal frequency), which was thought to reflect a longer integration window. In contrast, the expectation of a fast presentation rate induced less temporal integration, which was thought to reflect a shorter integration window. The observed changes in behavior were thus interpreted as evidence for adaptive control of integration.

Following these findings, the goal of the current study is to address whether individual AB task performance is related to the individual tendency to merge two events into one single representation, i.e., the amount of temporal integration. As assumed previously, the frequency of temporal integration is taken to reflect quite directly on the length of the temporal integration window. Based on our earlier findings [17], we hypothesize that individuals with a smaller AB will show less temporal integration, and thus, use a shorter temporal integration window than individuals with a larger AB. Furthermore, we aim to reveal the role of order reversals in relation to the occurrence of temporal integration. If order reversals in a paradigm using alphanumerical stimuli actually reflect temporal integration [18], then the positive relation between order reversals and AB magnitude as found earlier may be absent in a paradigm where temporal integration of stimuli is possible, i.e., using symbol stimuli: This is because the remaining order reversals that are observed with such stimuli should reflect ‘true’ order problems, and no longer reflect integrations. In the current paradigm, these true order reversals can thus be measured directly.

## Methods

### Participants

The Psychology Ethical Committee of the University of Groningen approved the study, and participants signed a written informed consent form before onset of the experiment. A total of 100 students participated, after which they received course credits in return. The experiment was performed together with another experiment on individual differences that is reported in [21]. The order of both experiments was counterbalanced, and together the experiments were completed in ~90 minutes. The current experiment took ~60 minutes. Nine individuals were excluded, because T1 accuracy was < 50%, or data logging did not succeed. This left 91 participants (55 women; mean age = 20.43 years, ranging 18–29) for the final analyses.

### Apparatus and stimuli

**Fig 1:**
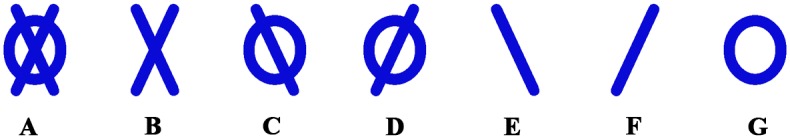
Target stimuli. The symbols that were used as target stimuli.

The task was performed using E-prime 2.0 software, and presented in the center of a 19-inch CRT monitor with a 100 Hz refresh rate. Target stimuli were blue symbols, as shown in Fig 1, whereas distractor stimuli were black, 52-point Courier New, uppercase consonants, excluding “Q”, “X”, and “Y”. The task was presented on a white background. The use of colored targets was motivated by earlier findings that task difficulty was too high when all stimuli were presented in black [18].

### Procedure

The task started with a practice block of 26 trials, which was followed by a test block of 528 trials. Each RSVP contained 19 stimuli, and was preceded by a 200-ms fixation cross. Stimulus presentation rate was 70 ms with an inter stimulus interval of 10 ms. In 504 of the 528 trials, two target symbols (Fig 1, symbols B-G) were presented, which could be visually combined into one symbol (symbols A-D). In 24 trials, only one target symbol was presented (symbols A-D). T1 was presented as either the fifth or the seventh stimulus in the stream. In dual-target trials, T2 was presented one, three, or eight serial positions after T1, at lag 1, 3, or 8, respectively. All lags were presented equally often. Per trial, distractor letters were pseudo-randomly selected under the constraint that successive distractor letters were never the same. In addition, target symbols were selected under the constraint that symbols were never identical within an RSVP, and that symbols with overlapping features were never presented as a pair, e.g., “**/**” was never presented in combination with “X”. Following each RSVP, participants were prompted to enter the symbols in the order they had seen them, using a stickered numeric keyboard. Participants could indicate whether they had seen a single symbol or no symbol by pressing the space bar.

### Statistical methods

Statistical analyses were performed using R (version 2.14.2; [22]), and the *lmerTest* package [23]. The data were analyzed using Generalized Linear Mixed Models (GLMM), where “participants” was added as random intercept to account for repeated measures.

## Results

### Accuracy

**Fig 2:**
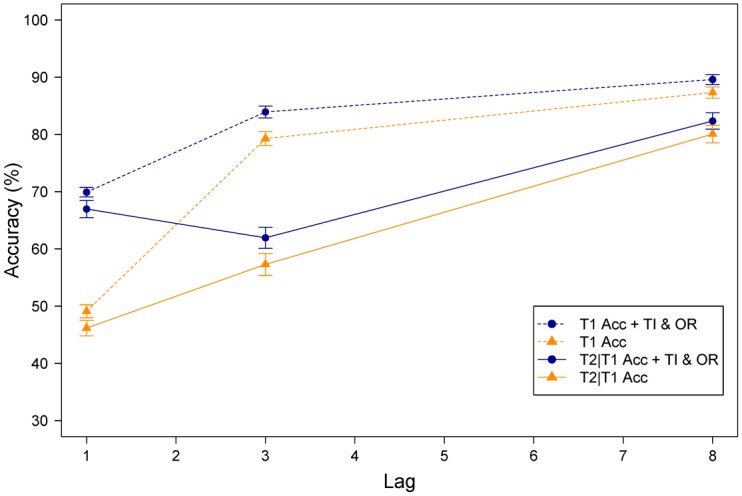
Task accuracy. Mean T1 accuracy and mean T2 accuracy given that T1 was identified correctly (T2|T1) without order reversal trials or temporal integration trials (dark yellow lines). Mean T1 and T2|T1 accuracy where both order reversals and temporal integration trials are counted as correct (blue lines). The error bars reflect the standard errors of the mean.

**Fig 3:**
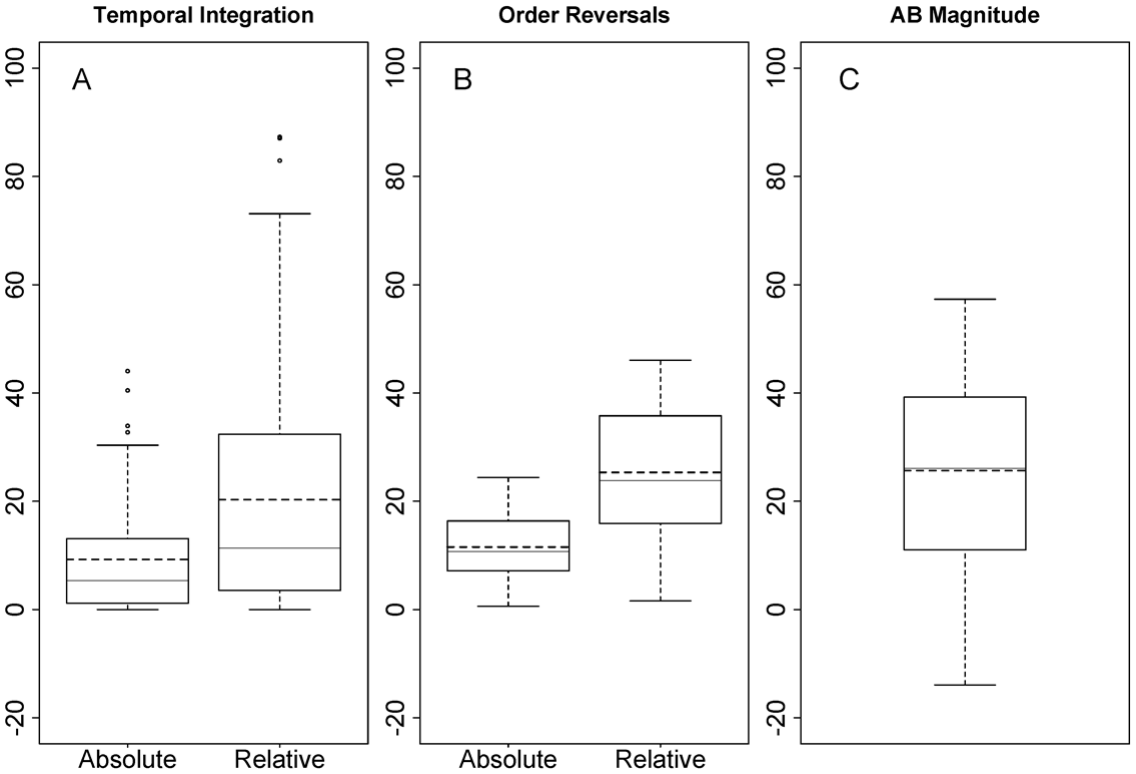
Distribution of individual differences. Boxplots depicting the distribution of individual differences for A) temporal integration at lag 1, B) order reversals at lag 1, and C) AB magnitude. For A) temporal integration and B) order reversals, the distribution is plotted for both the absolute and relative data. Relative is defined as trials where both target features are reported correctly, either in correct order, reversed order, or integrated. Per boxplot, the mean of the variable is indicated by the black dashed line, whereas the median is indicated by the light grey solid line.

Task performance is graphed in Fig 2. The dark yellow lines depict T1 and T2 given correct report of T1 (T2|T1) accuracy, without order reversal trials or temporal integration trials counted as correct, whereas the blue lines depict T1 and T2|T1 accuracy with both order reversals and temporal integration trials counted as correct. AB magnitude was calculated as T2|T1 accuracy at lag 3 relative to T2|T1 accuracy at lag 8 ((T2|T1_lag8_ –T2|T1_lag3_)/ T2|T1_lag8_), and ranged from -13.91% to 57.32% (mean = 25.66, *SE* = .13). In Fig 3A–3C, we graphed the distribution of individual differences regarding temporal integration at lag 1 (A), order reversals at lag 1 (B), and AB magnitude (C). In Fig 3A and 3B, the distribution of order reversals and temporal integration are plotted for both the absolute and the relative data; the latter are defined as trials where both target features are reported correctly, either in correct order, reversed order, or integrated (i.e., the trials represented by the blue lines in Fig 2).

### Temporal integration

**Fig 4:**
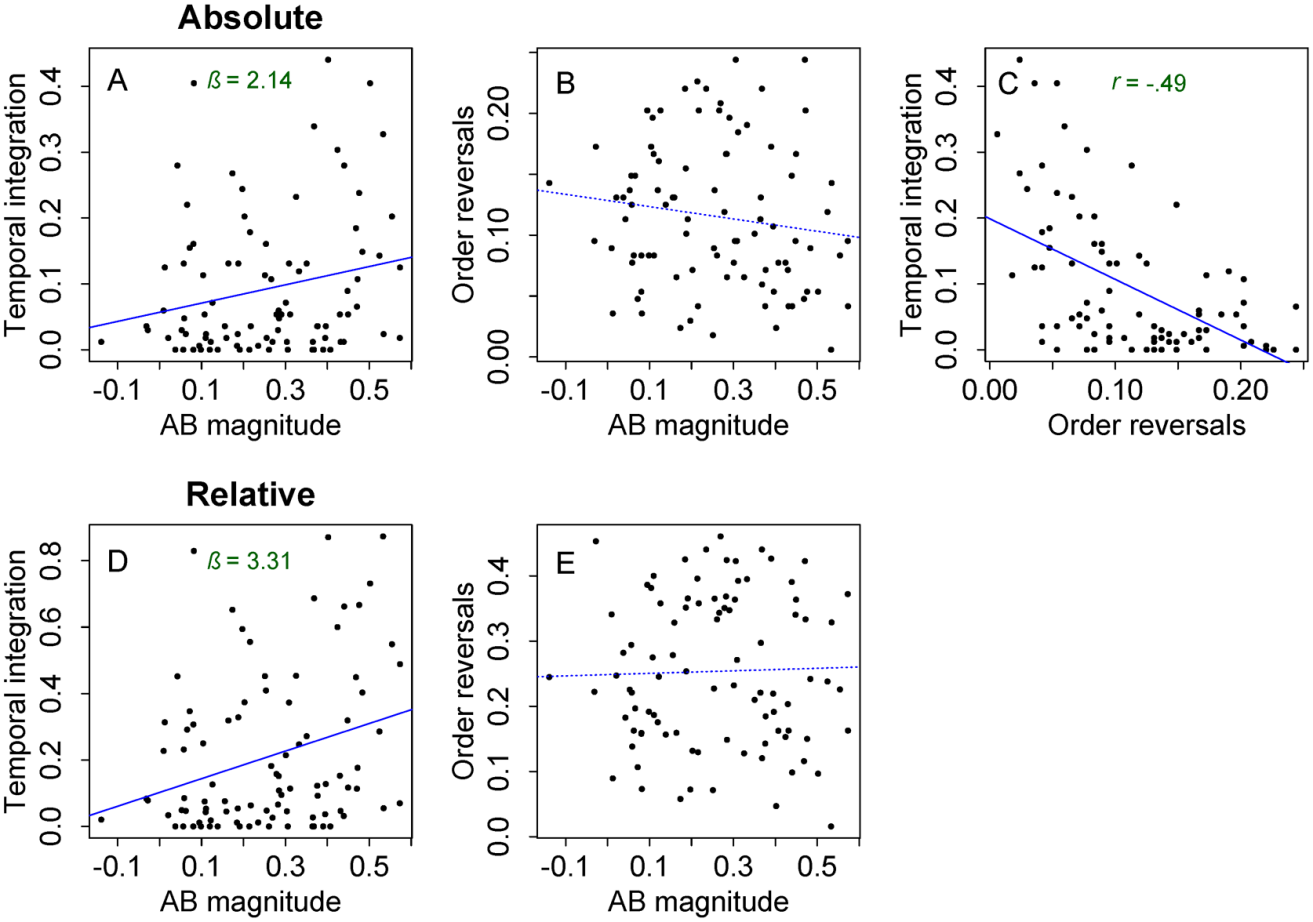
Relations between temporal integration, order reversals and AB magnitude. The relation between A) mean temporal integration and AB magnitude, B) mean order reversals and AB magnitude, and C) mean temporal integration and mean order reversals when taking all trials into account (A-C). The relation between D) mean temporal integration and AB magnitude and E) mean order reversals and AB magnitude in the relative data (D-E), defined as trials where both target features are reported correctly, either in correct order, reversed order, or integrated. For graphical purposes, per scatterplot a trend line was added depicting simple linear regression between the two given variables.

In 9.23% (*SE* = .23) of all lag-1 trials, the symbols presented as T1 and T2 were reported as the integrated symbol for T1, and T2 was reported as being absent. For lag 3 and 8 this occurred in only .88% (*SE* = .001) and .35% (*SE* < .001) of the trials, respectively. With a GLMM with AB magnitude as fixed factor, we found that the amount of integration in lag-1 trials was significantly related to AB magnitude, *β* = 2.14, *SE* = 1.04, *z* = 2.05, *p* = .040. When analyzing the relation between individual AB magnitude and temporal integration in lag-1 trials relative to task performance, the predictive effect of AB magnitude was even stronger, *β* = 3.31, *SE* = 1.23, *z* = 2.68, *p* = .007. Given the positive direction of this relationship, depicted in Fig 4A and 4D, this finding indicates that individuals with a larger AB have a higher tendency to integrate T1 and T2 at lag 1 into a single representation. In the relative data, integration occurred on 20.3% (*SE* = .48) of the lag-1 trials, 1.7% (*SE* = .14) of the lag-3 trials, and .47% (*SE* = .06) of the lag-8 trials.

### Order reversals

For lag-1 trials, the report order of both targets was reversed in 11.55% (*SE* = .26), 3.7% (*SE* = .15) for lag-3 trials, and 1.92% (*SE* = .11) for lag-8 trials. As tested with a GLMM, we found no effect of AB magnitude on the number of order reversals in lag-1 trials (*p* = .10; Fig 4B). There was also no effect of individual AB magnitude when analyzing the occurrence of order reversals in lag-1 trials relative to task performance (*p* = .10, Fig 4E). In the relative dataset, order reversals occurred on 25.33% (*SE* = .52), 7.1% (*SE* = .29), and 2.59% (*SE* = .15) of the lag-1 trials, lag-3 trials, and lag-8 trials, respectively.

As shown in Fig 4C, regarding lag-1 trials, we found a negative Spearman’s rank correlation between the mean number of order reversal trials and temporal integration trials (*r* = -.49, *p* < .001). This negative relation suggests that individuals who made fewer order reversals showed higher temporal integration and vice versa.

## Discussion

The aim of the current study was to investigate whether individual AB magnitude is related to the tendency to integrate two events that are in close temporal proximity into one event. Earlier work has shown a positive relation between AB magnitude and order reversals [17], which have been suggested to reflect the loss of temporal information [18]. Therefore, we expected temporal integration to occur more often for individuals with a larger AB, reflecting the use of a longer temporal integration window, than for individuals with a smaller AB. Furthermore, if order reversals reflect integration of the two targets in one visual event in classic alphanumeric AB tasks [18], the relation between order reversals and AB magnitude may not be present in a paradigm where temporal integration of stimuli is possible, i.e., in the current experiment.

As hypothesized, we found a positive relation between AB magnitude and the amount of temporal integration at lag 1, such that a smaller AB is associated with a lower tendency to integrate information into one visual event. This effect was even stronger when analyzing the amount of temporal integration relative to task performance. We found no evidence for a relation between true order reversals and AB magnitude in either the absolute or relative dataset. Finally, we found that the occurrence of order reversals was negatively related to the amount of temporal integration, such that individuals who made more order reversals showed less temporal integration.

### Temporal integration

The current findings confirm our hypothesis that individuals with higher AB task performance use a shorter temporal window to integrate information into one visual event, and thus, have a higher preservation of temporal information than individuals with lower AB task performance. This positive relation between temporal integration and AB magnitude fits with the distribution of erroneous responses in relation to individual AB task performance as described in [17]; using a paradigm with letter stimuli only, we have found that individuals with a smaller AB showed most delay—the center of mass of individual target report—at lag 1, after which the amount of delay diminished as a factor of time. In contrast, individuals with a larger AB showed the least delay at lag 1, after which delay increased as a factor of time. When assuming that these two opposite patterns reflect the length of the applied temporal integration window, it may be that individuals have most trouble identifying T2 when it is presented just after the window in which T1 is processed, i.e., for individuals with a larger AB at lag 3 and for individuals with a smaller AB at lag 1.

It is conceivable also that having a relatively long temporal integration window renders the task of identifying the targets more difficult, with increased AB magnitude as a result. Consider that at lags in which a distractor appears between targets, i.e., lag 2 and beyond, the perception of a target may suffer from the temporal proximity of the ensuing distractor. This effect may be compounded if the distractor is more likely to become part of the target event, a position from which it is likely to cause more interference. Such a scenario is more likely if an observer has the tendency to use (overly) long integration windows. Further down the line, the difficulties caused by integrated-distractor interference on T1 can affect T2 also by amplifying the attentional challenges, or in other words, the AB. Previous studies on the relation between target difficulty and the AB seem to support this idea [24–26].

In addition, given that the length of the integration window was found to be adaptable [19], the finding that AB task performance is related to the length of the temporal integration window is also in line with earlier findings that the size of the AB can be manipulated by an additional distracting task [5, 7, 8], or diminished by training [27–29]. Moreover, the current results indicate that individuals with a larger AB are less able to preserve episodic distinctiveness than individuals with a smaller AB, which actually goes against the idea that the AB occurs to preserve episodic distinctiveness, as proposed by the eSTST model [16].

Finally, it may be noted here that the current experiment did not produce lag-1 sparing, which was also observed in some of the experiments of similar design reported by Akyürek and colleagues [18]. Although identification performance at lag 1 is surely enhanced by temporal integration, this must be seen as an indirect relationship. If integration is relatively frequent in a given task, perhaps because of its particular target stimuli, it may very well also allow more opportunity for backward masking. This would counteract any positive contribution of temporal integration to the overall identification rate. Therefore, the occurrence or absence of sparing per se is not fully indicative of the processes underlying performance at lag 1.

### Order reversals

In the current study, we found no relation between individual AB task performance and true order reversals. These results are not in conflict with the suggestion of [18] that order reversals in a paradigm using alphanumerical stimuli reflect temporal integration of both targets in one visual event. That is, given that temporal integration was possible in the current experiment, it can be assumed that order reversals in this paradigm actually reflect true order errors rather than integration. Therefore, temporal integration trials probably took over at least part of the explained variance that is ascribed to order reversals in paradigms using alphanumerical stimuli. This can explain why we did not find a relation between order reversals and AB magnitude in the current study, whereas we did find such a relation in earlier studies using alphanumerical stimuli [17, 21]. Furthermore, our finding that temporal integration occurred at lag 1 does not support accounts where order reversals are subscribed entirely to T2 entering working memory earlier than T1 due to a stronger representation and more rapid consolidation of T2 compared to T1 [16, 30, 31]. However, in contrast to [18], it must be noted that we still found a relatively high number of order reversals. In view of the collective evidence to date, it nonetheless does seem that temporal integration can explain a substantial part of the order reversals in more classic AB tasks, whereas true order reversals may still be explained by other aspects of information processing. One such account that has been advanced is that they result from prior entry, where stronger stimuli are perceived as having occurred first. In the AB task, this could be triggered by a transient attentional boost that is elicited by T1, which has been hypothesized to lag slightly and land on T2 when it arrives at lag 1 [16, 30, 31].

Regarding the observed negative relation between the number of order reversals and the amount of temporal integration, it seems that participants either integrated or reversed information. A confound regarding this negative correlation may be that these two types of answers exclude one another. However, as can be seen in Fig 3A and 3B, neither temporal integration, nor order reversal rates appear to be at ceiling level (reaching maxima of ~30–40% of the trials), and thus we think that the negative correlation can be assumed to be genuine.

In summary, we revealed that individuals with a smaller AB have a lower tendency to temporally integrate information into one visual representation when presented in direct succession. In addition, we found no relation between true order reversals at lag 1 and AB magnitude. Evidence for a trade-off between integrations and order reversals was found instead; individuals tended to either integrate or reverse information at lag 1. Following this, we conclude that individuals with a smaller AB use a shorter temporal integration window than individuals with a larger AB, and therefore have a higher preservation of temporal information. Furthermore, order reversals in classic AB paradigms seem to at least partially reflect temporal integration of both targets at lag 1, as was already suggested in [18]. Given the negative relation between temporal integration and order reversals observed in the current paradigm, these two patterns of behavior seem to be two sides of the same coin.

## Supporting Information

S1 File The collected data sample. (TXT)
